# Reduction of detection limits in monitoring of internal exposures by a combined evaluation of emissions and spectra

**DOI:** 10.1007/s00411-024-01079-y

**Published:** 2024-07-09

**Authors:** Oliver Meisenberg

**Affiliations:** Federal Office for Radiation Protection (BfS), Medical and Occupational Radiation Protection, 85764 Oberschleißheim, Germany

**Keywords:** Gamma spectrometry, Alpha spectrometry, ISO 11929, Internal monitoring

## Abstract

Routine monitoring of internal exposures requires the detection of effective doses of at most 1 mSv per calendar year. For some radionuclides, this requirement cannot be satisfied by a conventional evaluation of the spectra that are gained in alpha or gamma spectrometry. However, since several measurements are conducted per calendar year on a regular basis, a combined evaluation of measurements, i.e. the evaluation of sum spectra, is possible. Additionally, radionuclides that feature several emissions of alpha or gamma radiation allow a combined evaluation of their emissions. Both methods can lead to significantly smaller detection limits as compared to a separate evaluation of spectra in many cases. However, the variation of parameters that influence the evaluation such as the measurement efficiency, abundance and chemical yield requires specific calculations and treatments of the spectra as well as a manipulation of the channel contents: In a combination of emissions, energy regions are summed and evaluated with a combined efficiency that is weighted by the abundances. In a combination of spectra, the channel contents must be scaled by the ratio of the calibration factors before the summation of the spectra. In the routine monitoring of short-lived radionuclides that feature a variety of emissions such as ^225^Ac, these combinations are particularly effective in reducing the detectable annual effective dose. For alpha spectrometry of ^225^Ac, both methods applied together can lead to a detectable effective dose of about 1 mSv per year as compared to a dose of about 90 mSv with a conventional separate evaluation.

## Introduction

Routine internal monitoring is an important contribution to the radiological monitoring of workers that handle unsealed radioactive sources and feature a risk of intakes of radioactivity into the body. It comprises methodologies of in-vivo measurements with whole-body and partial-body counters, in-vitro bioassays of urine and faeces and (not dealt with in this study) measurements of the airborne radionuclide concentration at the workplace. In contrast to most other applications of radiation measurements, in routine internal monitoring the sum of the activities from all measurements within a period of time (typically a calendar year) is meaningful, as described below in the generalisation of Eq. 2. Therefore, a combined evaluation of all measurements during that period of time can be conducted, leading to a smaller detection limit (as defined in ISO 11929-1; ISO 2019) than the one from a separate evaluation of each single measurement.

Metrological methods that are applied in routine internal monitoring comprise in particular gamma spectrometry for in-vivo measurements and alpha spectrometry and liquid scintillation counting (LSC) for in-vitro bioassays. International standards and recommendations account for a consistent implementation in various countries, resulting in uniform detection limits that need to be achieved and monitoring intervals (Etherington et al. 2018; ISO 2006).

Regarding the detection limit and the monitoring interval of routine internal monitoring, 2 inequalities depending on properties of the monitored radionuclide must be satisfied:

1 $$m\!\left({\Delta }t/2\right)\!/m\!\left({\Delta }t\right)\le 3$$

2 $$n\,e\,\text{DL}/m\!\left({\Delta }t\right)\le 1\,\text{mSv}$$

where *m*(*t*) is the value of the bioassay function (retention or excretion) at time *t* between intake and measurement (for in-vivo monitoring) or sampling (for in-vitro monitoring),

Δ*t* is the monitoring interval,

$n=\text{365 d}/{\Delta }t$ is the number of measurements per year,

*e* is the effective dose coefficient of the radionuclide and DL is the detection limit of the activity in the body or the bioassay sample.

Equation 1 depends only on the fixed biokinetic data of the monitored radionuclide, not on the detection limit. It ensures that a standard evaluation, which assumes an intake at the middle of the monitoring interval (period of Δ*t*/2 before the measurement or sampling), underestimates the intake by not more than a factor of 3 as compared to the maximal possible intake at the beginning of the monitoring interval (Δ*t* before the measurement or sampling). Equation 1 leads to a variety of possible values of monitoring intervals for the respective radionuclide, limited by international standards to a range from 7 to 180 days.

Equation 2 ensures that summed over the results of all separately evaluated measurements and subsequent dose calculations in a year, an effective dose of 1 mSv can be detected. It states a requirement for the detection limit to be achieved so that at least for one of the different possible values of monitoring intervals from Eq. 1, also Eq. 2 is satisfied and the applied metrological method is regarded as applicable. Equation 2 is only valid if the detection limits of all measurements in a monitoring interval are equal; if they differ from each other, the factor *n* needs to be replaced by a summation over the contributions from the single measurements.

For different radionuclides, the satisfaction of Eqs. 1 and 2 poses a requirement for the detection limit of different challenge. In particular, radionuclides with comparably great dose coefficients *e* and small monitoring intervals Δ*t* require comparably small detection limits that can typically not be achieved. Such radionuclides comprise in particular short-lived alpha emitters such as ^225^Ac, which has recently gained high relevance for the production of therapeutic radiopharmaceuticals (Eychenne et al. 2021; half-life 9.9 d, effective dose coefficient for inhalation for default type M 1.8·10^− 6^ Sv/Bq, possible monitoring intervals according to Eq. 1 for whole-body counting 7, 14 and 30 days, for urine analysis 7 days; Paquet et al. 2019).

Additionally, multi-emission radionuclides feature emissions that are distributed over several energies and that are evaluated separately in conventional methods. The information that is contained in the several emissions together is capable of reducing the detection limit as compared to a separate evaluation of the emissions.

In the following, 2 similar methods based on the summation of several emissions in single spectra (method a) and on the summation of spectra (method b), which result in a reduced detectable effective dose over a period of time, are presented. Both methods require a correct treatment of the uncertainties, some of which are uncorrelated whereas some others are correlated. Neither of these methodologies is applied in standard gamma-spectrometry software such as Genie 2000 (Mirion, USA), where the detection limit of a nuclide activity equals the smallest detection limit of the evaluated emissions (Mirion Technologies (Canberra) 2023), and GammaVision (Ortec, USA), where the detection limit of a nuclide activity is the mean of the detection limits of the evaluated emissions weighted by their abundances (Advanced Measurement Technology 2020). However, publications that present similar, yet slightly different methods for a combined evaluation of emissions (Korun et al. 2018) and spectra (Barker et al. 2005; Vivier et al. 2012) exist.

The 2 methods can be applied individually or jointly. Both methods ensure the satisfaction of the ideas behind Eqs. 1 and 2 but pose significantly less demanding requirements regarding the detection limit. Therefore, they are meant to be applied typically on measurements in which no activity was detected with a conventional separate evaluation. After the application of the methods, it is possible that still no activity is detected but compliance with the 1 mSv criterion from Eq. 2 is achieved or that activity is detected, which enables a dose calculation, statistical evaluation etc. in cases where this would not be possible with a conventional evaluation.

In the proofs of concept it is shown that the methodology is applicable and yields correct results. The implications on internal monitoring are shown in 2 practical examples where the application of the combined evaluation is compared with that of the conventional separate evaluation. The required steps of spectrum manipulations are presented for the Genie 2000 software by Mirion Technologies (USA) in Appendices A and B.

## Methodology

### Common aspects for both methods

In routine internal monitoring on regular intervals, the effective dose *E* caused by the intake of a specific radionuclide over a specific period of time, typically a calendar year, is calculated under conservative assumptions as follows:

3 $$E=\sum _{i}{E}_{i}=\sum _{i}{A}_{i}e/m\!\left({\Delta }t\right)=e/m\!\left({\Delta }t\right)\sum _{i}{A}_{i}={A}_{\text{tot}}e/m\!\left({\Delta }t\right)$$

where the index *i* denotes assessments from individual regular measurements and *A*_tot_ is the total activity in the body or in bioassay samples over the monitoring period.

Thus, the following equation is suitable to replace Eq. 2 in order to prove that an effective dose of 1 mSv in a calendar year is not exceeded:

4 $$E={\text{DL}}_{{A}_{\text{tot}}}e/m\!\left({\Delta }t\right)\le 1 \text{mSv}$$

where ${\text{DL}}_{{A}_{\text{tot}}}$ is the detection limit of a combined evaluation of the total activity in the body or bioassay samples from all single assessments over the calendar year.

The decision threshold decreases with increasing measuring time *T* at best by a factor of $1/\sqrt{T}$, equivalent to a factor of $1/\sqrt{n}$ at best if *n* measurements with equal measurement time are combined (according to Eq. D.11 in ISO 11929-1, example in Chap. 6 of ISO 11929-4 for different measurement times *t*_g_ and *t*_g_<*t*_0_; ISO 2019, 2020). The detection limit can decrease at best even by a factor of $1/T$ and $1/n$, depending on the parameters of evaluation (Eq. D.14 in ISO 11929-1).

In a conventional separate evaluation, the detection limit is required to ensure a detectable effective dose of each single measurement of $1/n$ mSv according to Eq. 2. With either of the 2 methods of a combined evaluation, the requirements regarding the detection limits of the single measurements are less restrictive. In the optimal case, where the detection limit decreases with $1/n$ when *n* measurements are combined, it would be sufficient to detect an effective dose of 1 mSv in each single measurement that is taken into account for the combined evaluation. The exact calculation depends on the calculation of the detection limit in the specific case and the general requirement from Eq. 4 holds in any case.

To evaluate the manipulated spectra in software, it is required to set up the usual model of evaluation with one single efficiency and one single abundance. Therefore, the correct calculation of these parameters will be a focus of the description of the methods.

### Realisation in the Genie 2000 software

All analyses of alpha and gamma spectrometry were conducted using the Genie 2000 software, version 3.4 (Mirion Technologies, USA). The general correctness of calculations of decision thresholds and detection limits according to ISO ﻿11929 in this software was verified by (Arndt et al. 2023). Manipulations of spectra in the CAM file format were conducted using the batch commands (Canberra Industries 2009), which are part of the Genie 2000 installation and which were called from a Python 3 script.

### Combination of emissions (method a)

In this method, the channel contents of all energy regions where emissions of the radionuclide of interest are expected, including a sufficient number of additional channels on both sides of the energy region, are summed. This is done by manipulating the channel contents and creating a new spectrum that contains the manipulated channel contents in one single, combined energy region (in this case that of the lowest-energy emission 1).

For the combined evaluation of emissions, the efficiency *η*_combined_ of the combined emission must be calculated from the efficiencies and abundances of the single emissions. In a separate evaluation of the emissions, the activity is calculated with an equal value from each single emission:

5 $$a=\frac{{c}_{1}}{T{\eta }_{1}{I}_{1}}=\dots =\frac{{c}_{n}}{T{\eta }_{n}{I}_{n}}$$

On the other hand, the same activity can be calculated from the combination of emissions, where the number of counts equals the sum of the counts of the single emissions, if the parameters of the evaluation are chosen correctly:

6 $$a=\frac{{c}_{1}+\dots +{c}_{n}}{T{\eta }_{\text{combined}}{I}_{1}}$$

Therefore, the combined emission can be evaluated with abundance *I*_1_ of the first emission and a suitably calculated efficiency:

7 $${\eta }_{\text{combined}}={\eta }_{1}+\frac{{I}_{2}}{{I}_{1}}{\eta }_{2}+\dots +\frac{{I}_{n}}{{I}_{1}}{\eta }_{n}$$

where:

*a* is the activity,

*c*_*i*_ is the number of counts of emission *i* (detected above the decision threshold or hypothetical below the decision threshold),

*T* is the measurement time,

*I*_i_ is the abundance of emission *i*,

*I*_1_ in particular is the abundance which is applied for the combined evaluation since all emissions are combined at the energy of emission 1 and.

*η*_*i*_ is the efficiency at the energy of emission *i*.

For the calculation of the uncertainty it must be taken into account that the efficiencies are typically strongly or even fully correlated because they typically originate from the same calibration. Therefore, for the calculation of the uncertainty, ${\eta }_{i}$ should be written as ${\eta }_{i\text{, rel}}\cdot {\eta }_{1}$ in Eq. 7 with ${\eta }_{i\text{, rel}}={\eta }_{i}/{\eta }_{\text{1}}$. Under best circumstances when the relative uncertainties of *η*_*i*_ and *η*_1_ are equal, the uncertainty of *η*_*i*, rel_ vanishes (cf. Appendix C) so that *η*_*i*, rel_ can be treated as an exact quantity without uncertainty:

8 $${\eta }_{\text{combined}}={\eta }_{1}+\frac{{I}_{2}}{{I}_{1}}{\eta }_{2\text{, rel}}{\eta }_{1}+\dots +\frac{{I}_{n}}{{I}_{1}}{\eta }_{n\text{, rel}}{\eta }_{1}$$

Whereas a consideration of small remaining uncertainties of *η*_*i*, rel_, when the relative uncertainties of *η*_*i*_ are slightly different, are beyond the scope of a manual calculation, computer software should not have problems with the exact uncertainty propagation of Eq. C.5.

Additionally, it is beneficial to compute the uncertainty of *η*_combined_*I*_1_ instead of those of the individual 2 quantities due to their correlation:

9 $$\begin{aligned} & u\!\left( {{\eta _{{\text{combined}}}}{I_1}} \right) \\ & \quad = \;u\!\left( {{\eta _1}{I_1} + {\eta _1}{\eta _{2{\text{, rel}}}}{I_2} + \ldots + {\eta _1}{\eta _{n{\text{, rel}}}}{I_n}} \right) \\ & \quad = \sqrt {\begin{array}{l}{u^2}\!\left( {{\eta _1}} \right){{\left( {{I_1} + {\eta _{2{\text{, rel}}}}{I_2} + \ldots + {\eta _{n{\text{, rel}}}}{I_n}} \right)}^2} \\ \quad + \eta _1^2{u^2}\!\left( {{I_1}} \right) + \eta _1^2\eta _{2{\text{, rel}}}^2{u^2}\!\left( {{I_2}} \right) + \ldots + \eta _1^2\eta _{n{\text{, rel}}}^2{u^2}\!\left( {{I_n}} \right)\end{array}} \end{aligned}$$

Since the evaluation software treats these 2 uncertainties as uncorrelated, the uncertainty of *η*_combined_ can subsequently be entered into the software by applying the equation of uncertainty propagation for the product of 2 uncorrelated quantities: ${u}_{\text{rel}}^{2}\!\left({\eta }_{\text{combined}}\right)={u}_{\text{rel}}^{2}\!\left({\eta }_{\text{combined}}{I}_{1}\right)-{u}_{\text{rel}}^{2}\!\left({I}_{1}\right)$.

Whereas a similar combination can be applied to the abundance instead of the efficiency, it might be simpler to apply the combination to the efficiency according to Eq. 7 instead of to the abundances in cases where efficiencies and abundances vary. This is because the efficiency can easily be incorporated into the automated evaluation through a Genie 2000 batch command whereas the abundance is read from an entry in the nuclide library. A counterexample is alpha spectrometry as shown below.

In the resulting spectrum, only the combined emission is evaluated whereas the original emissions of the nuclide of interest are disregarded.

### Combination of spectra (method b)

In this method, all spectra from the *n* measurements of a monitored person within a period of time up to one year are summed. This leads to a single spectrum as if one single hypothetical measurement of the individual with his or her variable body activity of radionuclides was conducted (in in-vivo monitoring) or one single average bioassay sample (in in-vitro bioassay) was taken distributed over the course of the year with a measurement time that equals *n* times the measurement time of a single measurement.

In contrast to the combination of several emissions within a single measurement, the real activities (if detected or not) over several measurements can vary. Therefore, Eqs. 5 and 6 are not applicable for the combination of spectra. Instead, the arithmetic mean of the activities of the measurements, which might feature different measurement times *T*_*i*_ and overall efficiencies *η*_*i*_ (including not only the counting efficiency, but in alpha spectrometry also the aliquot, the chemical yield and the decay factor) is calculated as

10 $$\eqalign{& \mathop a\limits^ - = \frac{1}{n}\left( {\frac{{{c_1}}}{{{T_1}{\eta _1}I}} + \ldots + \frac{{{c_n}}}{{{T_n}{\eta _n}I}}} \right) = \cr & \frac{1}{n}\left( {\frac{{{c_1}}}{{{T_1}{\eta _1}I}} + \ldots + \frac{{{c_n} \cdot \left( {{T_1}{\eta _1}} \right)/\left( {{T_n}{\eta _n}} \right)}}{{{T_1}{\eta _1}I}}} \right) = \cr & \frac{{{c_1} + \ldots + {c_n} \cdot \left( {{T_1}{\eta _1}} \right)/\left( {{T_n}{\eta _n}} \right)}}{{n{T_1}{\eta _1}I}} \cr}$$.

In Eq. 10, the measurement with the smallest *T*_*i*_*η*_*i*_ must stay unchanged and the numbers of counts *c*_*i*_ of all other measurements must be scaled downwards so that no unfounded statistical precision is pretended. Since this calculation is conducted with spectra in which no activity was detected, it cannot be conducted arithmetically on the numbers of counts after the evaluation of spectra but the spectra must be manipulated and scaled. Besides, it must be noted that simply adjusting the denominators in Eq. 10 (the individual measurement times or efficiencies) while keeping the numbers of counts in the numerator unchanged does not lead to exact results.

Of each kind of parameter with index *i* from Eq. 10, the numbers of counts *c*_*i*_ and measurement times *T*_*i*_ are uncorrelated between the single measurements. Of the parameters that constitute the overall efficiencies *η*_*i*_, the counting efficiencies are identical in many cases (when the same calibration was applied), however in other cases they can be strongly correlated (when a new calibration with the same standard was conducted) or uncorrelated (when a new calibration with a different standard was conducted). Aliquots, chemical yields and decay factors are uncorrelated between the single measurements. For the treatment of the uncertainty, it is assumed that the counting efficiencies are identical between the combined measurements so that ${\eta }_{i}/{\eta }_{n}$ reduces to the ratio of the uncorrelated quantities (aliquots, chemical yields, decay factors), which allows a simple calculation of the combined uncertainty in Eq. 10 without taking into account correlations. In cases when they are strongly correlated, the same substitution with their ratio as an exact value as in Eq. 9 can be conducted.

In some cases, a set of measurements with small detection limits must be combined with a set of other measurements with large detection limits to cover the monitoring period. In these cases, it is possible that this will increase the detection limit compared to that of the combination of the measurements with small detection limits only. Example are presented in the Results, e.g. in *Gamma spectrometry: proof of concept using*^*60*^*Co*. This phenomenon is not specific to the combined evaluation of spectra. It corresponds to the fact that a measurement with large detection limit consumes a large amount of the 1 mSv criterion in the generalised version of Eq. 2 at the expense of the detection limits of all other measurements in an individual evaluation of spectra.

Because common spectrometry software treats channel contents only as integer numbers, it must be checked after the scaling of a spectrum if rounding errors have caused a deviation from the calculated number of counts. When this happens, the required amount of counts must be added to channels in the region of interest or subtracted from them in a second step after the scaling.

In cases where a background must be subtracted, it can be necessary to increase the background measuring time so that it exceeds the total measuring time of the combined measurement. Otherwise the combination method may not improve the detection limit of the combined measurement significantly (example in Chapter 6 of ISO 11929-4, Eq. 14 for *t*_g_≫*t*_0_; ISO 2020).

Background corrections shall be done with an overall background after the combination of spectra instead of for each single spectrum. This is because channel contents corrected for a background can become negative due to the uncertainty of counts and many analysis software tools do not process negative numbers of counts. The background spectra must be treated with the same methodology of a combination of emissions and/or spectra as the spectra from the measurements.

### Specific features of alpha spectrometry

Since in alpha spectrometry the efficiency is constant over the measured energy range, Eq. 7 can easily be replaced by a summation of the abundances: ${I}_{\text{combined}}={I}_{1}+\dots +{I}_{n}$.

Whereas gamma spectrometry depends on the detection of Gaussian-shaped peaks, analysis in alpha spectrometry works with fixed regions of interest (ROI). In cases where the ROIs of the separate emissions are adjacent to each other, the combination of emissions (method a) does not require a manipulation of the spectrum but can be simplified to one ROI covering the energy range of all emissions. In Genie 2000 this can be achieved by a library peak search, where the nuclide library contains one combined entry with the mean energy of the combined ROI and a suitable setting of the ROI width.

In the combination of spectra (method b), typically more differences in the parameters of the evaluation must be considered in alpha spectrometry of biological samples as compared to whole-body counting though, in particular different aliquots, chemical yields and decay factors.

## Results

### Alpha spectrometry: proof of concept using ^225^Ac

2 samples (A and B) of an aqueous solution of ^225^Ac were provided by a manufacturer of radiopharmaceuticals (ITM, Germany). The activity was certified by the provider without traceability. Details of the chemical separation and measurement are described in Hartmann et al. (2024). Measurement times were 20,000 s, the background measurement times were 300,000 s. Parameters of the evaluation of the measurement results differed between the 2 samples: aliquot, decay factor, chemical yield and counting efficiency. For the sake of simplicity of the evaluation and the manipulation of the spectra for the combined evaluation, all these parameters were combined in the overall efficiency of the measurement (with their combined uncertainty) while all other parameters were set to 1 (with uncertainty 0). These overall efficiencies were 13.8% for B and 4.5% for A (0.329 of B) with a combined relative uncertainty of 12%.

^225^Ac and its decay products in secular equilibrium feature alpha-particle emissions in the following 4 energy regions (in keV): 5610–5830 (^225^Ac), 6130–6340 (^221^Fr), 7070 (^217^At), 8380 (^213^Po). All abundances, disregarding negligible differences, are 100%. The emission at 8380 keV was not evaluated because it lies outside the energy region of the measurement device, resulting in 3 evaluated emissions. As it is frequent in alpha spectrometry when nuclides in secular equilibrium contribute to the emissions, both the abundances and the efficiencies of all emissions were equal, making the combination of emissions trivial and yielding either an efficiency of 3 times the efficiency of a single emission or an abundance of 300%. The 3 emissions in each of the 2 spectra were combined (method a). As explained above, this was also conducted with the background spectra.

For the combination of the 2 spectra (method b), the spectrum with the smaller overall efficiency (A) was kept as it was whereas the other spectrum was scaled by a factor of 0.329 equalling the ratio of the efficiencies before adding the 2 spectra. The overall efficiency for the evaluation of the combined spectrum equalled that of A, the measurement time was twice that of spectrum A. This was done without previous combination of the emissions (method b only) and after combination of the emissions as a joint application of the 2 methods (method a & b).

The results and characteristic limits of the separate measurements and of the different combinations are presented in Table 1.

**Table 1:** Calculated activities and characteristic limits for a separate evaluation and different combined evaluations of the 2 alpha-spectrometry measurements with which the correctness of the method was tested. Without a combination of emissions, the presented activity is the arithmetic mean of the activities of the 3 emissions, the uncertainty is that of this mean and the decision threshold and detection limit is the minimum of those of the single emissions as it was reported by Genie 2000. All uncertainties are presented with a coverage factor *k* = 2

Method	Spectrum	Activity (Bq)	Uncertainty (Bq)	Decision threshold (Bq)	Detection limit (Bq)
Separate evaluation	A	91	18	0.84	4.8
	B	98	15	0.28	1.6
Method a: Combination of emissions	A	93	24	0.48	2.0
	B	97	21	0.092	0.52
Method b: Combination of spectra	A + B	93	15	0.74	3.0
Method a & b: Combination of emissions and spectra	A + B	99	23	0.30	1.1

### Alpha spectrometry: application on urine measurements of ^225^Ac

For the internal monitoring of a worker that was exposed to ^225^Ac over a restricted period of time, 6 urine samples were taken (Hartmann et al. 2024). The chemical separation and measurement methodology were the same as in the proof of concept. The calibration factors for the calculation of the urine activity at the end of the sampling from the count rate varied among the measurements because of different aliquots (18–48%), decay factors (0.75–0.87), chemical yields (4–58%) and counting efficiencies (24.3–25.5%), in total leading to calibration factors from 35 to 600 Bq/cps (difference of a factor 17). Again, this resulted in significantly different detection limits of the single measurements. The uncertainties of the overall efficiencies (again including all mentioned parameters in a single efficiency) were about 12%.

Applying a conventional separate evaluation, an effective dose of 92 mSv extrapolated to one year could be detected (assuming an intake 7 days before sampling as in Eq. 2 of ISO 20﻿553; ISO 2006). If it was assumed that the highest observed chemical yields could be reliably achieved for all samples (whereas in reality they were achieved only for few samples), the detectable effective dose could be reduced to 26 mSv as presented in Hartmann et al. (2024). Both is far above the required limit of 1 mSv.

For a combined evaluation, (method a) the 3 emissions within each spectrum, (method b) the 6 spectra with their separate emissions and (method a & b) both the 6 spectra and their 3 emissions were combined. The achieved detection limits of the activity in the 24-hour urine samples and the detectable effective dose are presented in Table 2.

**Table 2:** Achieved detection limits and detectable effective doses for a separate evaluation and different combined evaluations of the 6 alpha-spectrometry measurements of urine of a worker that was exposed to ^225^Ac

Method	Spectrum	Detection limit (mBq)	Detectable dose per measurement (mSv)	Detectable dose in a year (mSv)^1^
Separate evaluation	1	51	2.3	
	2	8.3	0.37	
	3	8.1	0.36	
	4	140	6.1	
	5	23	1.0	
	6	9.4	0.4	92
Method a: Combination of emissions	1	22	1.0	
	2	3.5	0.16	
	3	3.4	0.15	
	4	57	2.6	
	5	9.7	0.43	
	6	4.0	0.18	39
Method b: Combination of spectra	1–6	32	1.4	75^2^
	6x spectrum 3 (hypothetical)	1.9	0.086	4.5^2^
Method a & b: Combination of emissions and spectra	1–6	14	0.65	34^2^
	6x spectrum 3 (hypothetical)	0.86	0.039	2.0^2^

^1^ Extrapolated to one year, assuming that each of the 6 measurements is representative for 1/6 of the 52 weekly measurements within the year

^2^ Meaning that of the 52 measurements in the course of a year the measurements in sets of 6 measurements each are combined

### Gamma spectrometry: proof of concept using ^60^Co

A sample of ^60^Co (activity 9.8 ± 0.4 Bq) was placed on an HPGe gamma-spectrometry detector. ^60^Co features 2 gamma-radiation emissions: at 1173 keV (abundance 99.85%) and at 1332 keV (99.98%). The sample was placed at 2 different differences (A and B), leading to efficiencies of the 2 emissions of 13.6% and 10.4% (A) and 8.2% and 6.5% (B). 10 measurements were conducted at each of these 2 setups for 20 min each.

First, measurements no. 1 of A and of B were evaluated with separate emissions. Second, these 2 measurements were evaluated with combined emissions (method a). The combined emissions were evaluated with a combined efficiency of 24.0% (A) and 14.7% (B) and with the abundance of the 1173 keV emission. Then, measurements from no. 1 up to no. *i* (*i* = 2–10) of A and of B were combined (method a & b with up to 10 spectra). Last, the 2 combinations of all 10 spectra of A and of B were combined with each other, leading to a combination of 20 spectra, 10 with efficiency A and 10 with efficiency B (method a & b with 20 spectra). For this purpose, the combined spectrum of A was scaled by a factor 24.0/14.7 = 0.61. A subtraction of a background was not required because of the lack of occurrence of ^60^Co in the background but the detection limit was strongly influenced by the continuum, which varied between the measurements (ca. 2–4 counts per channel).

**Fig. 1:**
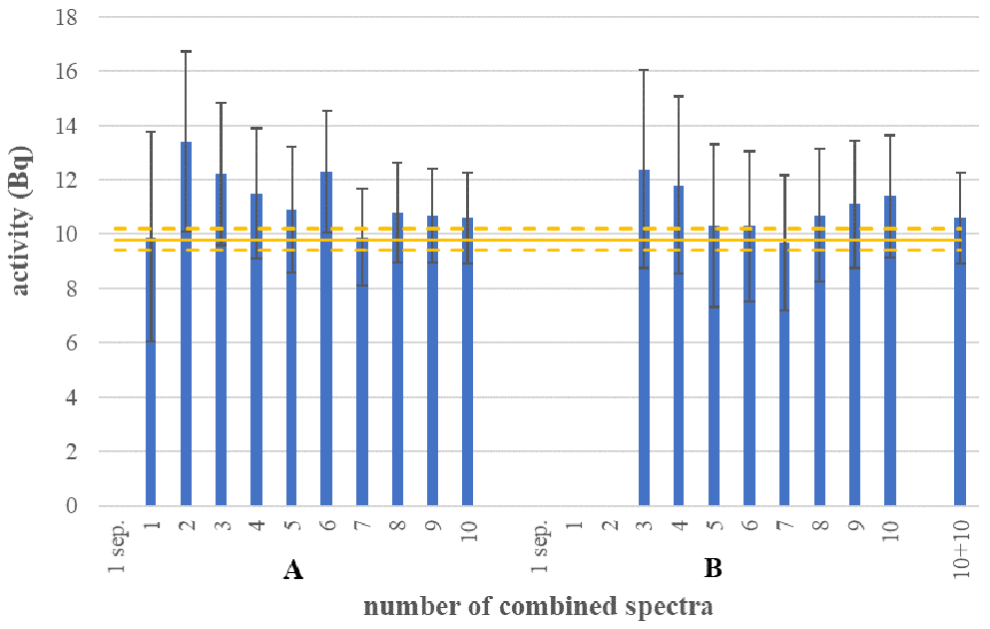
Blue: Activities determined in a separate evaluation of emissions, a combined evaluation of emissions (method a) and increasing number of spectra (1–10, method a & b) separately for the 2 groups and a combined evaluation of all 20 spectra, together with their uncertainties (coverage factor *k* = 2). Missing columns: ^60^Co not detected. Orange: True activity and uncertainty of the sample

**Fig. 2:**
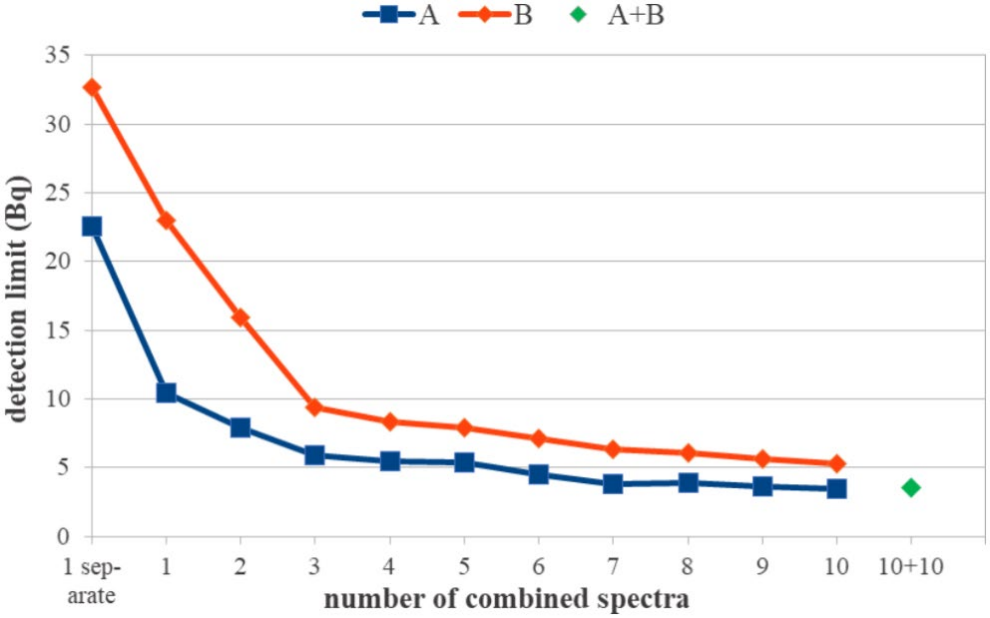
Detection limits for a separate evaluation of emissions, a combined evaluation of emissions (method a) and increasing number of spectra (1–10, method a & b) separately for the 2 groups and a combined evaluation of all 20 spectra. Lines are to guide the eye

The determined activities are presented in Fig. 1 and the detection limits are presented in Fig. 2.

### Gamma spectrometry: application on whole-body counting of ^225^Ac

Whole-body measurements of a person were conducted on a monthly basis, as it is acceptable for ^225^Ac, over the course of a year. 7 measurements were conducted with 4 detectors (M1-M7). Due to the failure of one of the 4 detectors, 5 measurements were conducted with only 3 detectors (M8-M12). It is assumed that the efficiencies of both types of measurements are uncorrelated because they were gained in different calibrations. All measurement times were 1200 s.

For the combined evaluation, the gamma emissions from the ^225^Ac decay chain at 218 keV (abundance 11.4%, efficiency in M1-M7 0.266%, in M8-M12 0.240%, i.e. 90.2% of M1-M7) and at 440 keV (26.1%, 0.223%, 0.193%, i.e. 86.3% of M1-M7) were applied. In a first step the 2 emissions were combined in the single spectra (method a): combination at 218 keV, abundance 11.4%, efficiency according to Eq. 7 (M1-M7: 0.777%, M8-M12: 0.682%), measurement time 1200 s. However, it was found that the detection limit was increased as compared to that at 440 keV only (Table 3). This is because of the significantly stronger continuum at 218 keV as compared to 440 keV (20 compared to 7 counts per channel), which impedes the more abundant emission at 440 keV when both energy regions are combined. Therefore, for the next steps spectra without a combination of emissions were applied (method b only).

The spectra were combined separately for M1+…+M7 and M8+…+M12: abundances and efficiencies as in the single spectra, measurement times 7·1200 s and 5·1200 s, respectively.

Lastly, the resulting 2 spectra were combined to achieve a combination of all spectra (M1+…+M12; method b only). This was done only for the emission at 440 keV because of the smaller detection limit; for this purpose, M1+…+M7 was scaled to 86.3% according to Eq. 10. The combined measurement time was 12·1200 s. The resulting detection limits and detectable effective doses are presented in Table 3.

**Table 3:** Achieved detection limits and detectable effective doses for a separate evaluation and different combined evaluations of ^225^Ac in 12 whole-body measurements over the course of a year

		Detection limit (Bq)	Detectable effective dose per measurement (mSv)	Detectable effective dose per year (mSv)
M1, …, M7	at 218 keV	270	73	-
	at 440 keV	83	23	280^1^
M8, …, M12	at 218 keV	320	88	-
	at 440 keV	96	27	320^1^
M1, …, M12	at 440 keV	-	-	300
Combination of emissions (method a):				
M1, …, M7		94	26	
M8, …, M12		97	26	
M1, …, M12		-	-	310
Combination of spectra (method b only):				
M1+…+M7	at 440 keV	30	8.2	98
M8+…+M12	at 440 keV	36	9.8	120
M1+…+M12	at 440 keV	25	6.8	81

^1^ Under the hypothetical assumption that 12 measurements were conducted under these conditions

## Discussion

### Alpha spectrometry: proof of concept using ^225^Ac

The activities of the spectra after the different methods of combination agreed with those of the original spectra, indicating that the methods use correct models of evaluation. The uncertainties after the combination of emissions (method a) are not smaller as compared to the separate evaluation; this is because also in the conventional separate evaluation the uncertainty is calculated as the uncertainty of the arithmetic mean of the results of all evaluated emissions. The uncertainty after combination of the spectra (method b) is slightly smaller than that of spectrum A and not smaller than that of spectrum B; this is because of the small contribution of spectrum B to the combined spectrum. However, the decision thresholds and detection limits are significantly reduced. The detection limits are reduced by a combination of emissions (method a) on average to relative values of 38% (1.13 times the optimal value of $1/n=$33% for *n* = 3 emissions). By a combination of the 2 spectra (method b), the detection limit was not reduced as compared to spectrum B with its great efficiency but it was reduced as compared to spectrum A with its small efficiency. As shown in the next example, this reduces the overall detection limit of a programme of routine internal monitoring over the course of the monitoring period.

### Alpha spectrometry: application on urine measurements of ^225^Ac

By a combination of emissions (method a), the detectable dose per year could be reduced to 42%; this is 1.3 times the optimal reduction of 1/3. The combination of the spectra without and with additional combination of emissions (methods b and a & b) did only slightly reduce the detection limits. This is because of the considerably different efficiencies of the single measurements and the required scaling of the spectra with better efficiencies. It was also tested which detection limits and detectable doses could be achieved if all measurements were conducted with the same efficiency as the best measurement (spectrum 3). Then a reduction to a value of 23% (1.4 times the optimal value of 1/6) could have been achieved as compared to a single evaluation of spectrum 3. With both methods combined, the reduction was to a value of 11% (1.9 times the optimal value of 1/3·1/6). A detectable dose of roughly 0.7 mSv can be estimated when this reduction is projected to a combination of all 52 measurements in a year and when all of these measurements are conducted with the same efficiency as spectrum 3.

Including those measurements that featured a high calibration factor (1 and 4) into the combination impaired the detection limit of the combined spectrum as compared to the other measurements. However, these measurements cannot be excluded in order to cover the entire monitoring period, during which such measurements with poor efficiency occurred. As explained in the *Combination of spectra* section, this problem also occurs also in a separate evaluation of a set of spectra, when some of them feature a high detection limit. The method of a combined evaluation of spectra is most efficient when all measurements feature similar efficiencies.

### Gamma spectrometry: proof of concept using ^60^Co

All activities that were determined comply with the true activity (including the uncertainty; Fig. 1). Those 2 whose uncertainty barely overlaps with the uncertainty of the reference activity (set A, 2 and 6 spectra combined) can be outliers with respect to the 95% confidence level of the uncertainty. The uncertainty can be seen to decrease with increasing number of combined measurements. Also in the combination of all 20 spectra, the determined activity complied with the true value despite the significant difference of the efficiencies of the 2 sets A and B. In set A, the combination of the 2 emissions led to a detection of ^60^Co in spectrum 1 (with an uncertainty of 39%) whereas ^60^Co was not detected with a separate evaluation of the emissions in the same spectrum. In set B, only the combination of 3 spectra (together with a combination of the emissions) led to a detection of the ^60^Co.

With the combination of emissions, a reduction of the detection limits close to the optimum of 1/2 was achieved with the spectrum from A and a reduction to 70% was achieved with the measurement from B (Fig. 2). The combination of an increasing number of measurements led to a decreasing detection limit (stronger or weaker from step to step when the continuum in the respective spectrum was smaller or greater). With 10 combined measurements, a reduction to 34% (A) and 23% (B) was achieved, the theoretical optimum is 1/10 = 10% for both sets. The exceedance of the optimum was smallest for the combination of 2 spectra and increased with increasing number of spectra. This is probably due to the increasing significance of the full correlation of the uncertainty of the efficiency throughout the combined spectra. The combination of the 10 measurements of A with those of B led to a slight increase as compared to A alone (to 101.7%) because of the smaller efficiency of B but to a strong decrease as compared to B alone (to 67%).

### Gamma spectrometry: application on whole-body counting of ^225^Ac

In none of the separate or combined measurements, ^225^Ac was detected. The detection limits show that even after combination of the 12 measurements, whole-body counting on monthly intervals is not sensitive enough to satisfy the 1 mSv criterion for routine internal monitoring (Table 3). However, the detectable dose was reduced from 300 mSv to 81 mSv, i.e. by a factor of 0.27. This is greater than the optimum of $1/12=0.083$ by a factor of 3.2.

## Conclusions

It was shown that the combination of emissions and spectra can yield significantly reduced detection limits and detectable effective doses in routine internal monitoring, for gamma and alpha spectrometry, also fulfilling the apparent prerequisite of yielding correct measurement results. The demand for these methods is restricted to cases where detectable effective doses are only barely above the required annual statutory limit of 1 mSv with a conventional separate evaluation. However, in such cases it would be negligent to disregard the reduction of the detectable dose that is possible with combined evaluations. The method of a combined evaluation of spectra (method b) is particularly efficient with short monitoring intervals, which lead to a large number of measurements per year. Specifically regarding ^225^Ac, alpha spectrometry can be made an acceptable means for routine monitoring with a detectable dose of less than 1 mSv per year when the potential of combining emissions (at best including all 4 emissions) and spectra is utilised, however only under the condition of reliably good efficiencies and in particular the chemical yield.

It was found that in some cases a combined evaluation of emissions and of spectra is not beneficial compared to a separate evaluation of those emissions and spectra with the smallest detection limits. Regarding the combination of emissions, the emissions with greater detection limits can be omitted in any case. Regarding the combination of spectra, the spectra from all measurements over the monitoring period must be taken into account, which leads to a smaller detection limit compared to a separate evaluation of all spectra in any case.

It is assumed that a similar reduction of the detection limit can be achieved in LSC measurements. However, the lack of functionality for the manipulation of raw data in common LSC software prevented an investigation.

In the conventional separate evaluation of spectra, the detection limit is required to be smaller according to Eq. 2 when many measurements are conducted in a year. This is also true in the case of a combined evaluation of spectra, though less distinct. Only in the theoretical optimal case of a reduction of the detection limit with inverse proportion to the number of combined spectra, the detection limit becomes independent from the number of measurements in a year and the required detectable effective dose is 1 mSv for each single measurement.

In the next time, it would be desirable if internal monitoring services could be granted the permission to apply the presented methodology on a case-by-case basis when they need to conduct routine monitoring where only this methodology provides a compliance with the requirements. This could serve as a basis for gaining further experience with the methodology in a variety of applications.

If the presented methodology proves to be applicable and helpful, the following provisions would be favourable in the long term:

- considering the methodology in international standards and recommendations for internal monitoring such as ISO 20553,
- explicating the calculation of decision thresholds and detection limits for a combined evaluation of emissions and spectra in ISO 11929, also considering different correlations between the parameters of evaluation,
- functionality for a user-guided combination of emissions and spectra in measurement software for alpha spectrometry, gamma spectrometry and LSC,
- in general a better functionality to manipulate the raw data in such software (scaling of spectra on the energy and the count scale, handling of non-integer and negative channel contents etc.).

The combined evaluation of emissions (method a) can be conducted in any case. However regarding the combined evaluation of spectra (method b), it must not be misconceived that this shall replace a separate evaluation. The combined evaluation shall rather complement the separate evaluation in the activity regime below the detection limit of the latter. In internal monitoring, a separate evaluation is still be needed for example in cases when remaining activity from an intake must be taken into account in the following measurements and for determining the point of time of an intake.

The scaling of spectra that is required in the described method of a combined evaluation of spectra (method b) when the efficiencies are different decreases the statistical information in these spectra. A better method of a combined evaluation of spectra without this weakness could exist but this is beyond the scope of this paper.

## Data Availability

The author declares that the data supporting the findings of this study are available within the paper.

## Appendix A: Combination of emissions in the Genie 2000 software

The commands from the Genie 2000 Batch Tools Support are described in Canberra Industries (2009), the CAM file parameters are described in Canberra Industries (2023).

The channel contents of the original spectrum were read as plain text using the following command:

report *filename_original* /template=c:\genie2k\ctlfiles\csv.tpl /section=channel_list /screen.

where *filename_original* is the path and filename of the original spectrum.

The calculation of the manipulated channel contents in the target ROI from those in the source ROIs sufficiently wide around the energy of the emissions were conducted in a Python script. For this purpose, the shape calibration, which contains parameters of the width of peaks, was read from the energy-calibration report, which was text-processed in a Python script:

report *filename* /template=c:\genie2k\ctlfiles\analysis.tpl /section=energycal /screen.

In this calculation, source ROIs were scaled along the energy axis (i.e. made wider or narrower) according to the ratio of the width at the target energy to that at the source energies according to the shape calibration. This was conducted by distributing the channel contents from a source ROI over the channel of the target ROI.

The spectrum with manipulated channel contents was generated in the Toolkit format, which is defined as a list of the channel contents as plain text. Subsequently, the spectrum was converted into the CAM format using the following command:

- filecnvt *filename_toolkit filename_cam* /toolkit.
- where *filename_toolkit* and *filename_cam* are arbitrary filenames of the manipulated spectrum in Toolkit and in CAM format, respectively.
- The settings of the original spectrum (energy and efficiency calibration, counting time etc.) were transferred to the manipulated spectrum using the following commands:
- strip *filename_original filename_copy* /factor=1.0.
- strip *filename_original filename_cam* /factor=–1.0.
- where *filename_copy* is the filename of a copy of the original spectrum.

The first command generates a spectrum with the original settings but all channels containing zero counts. The second command adds the manipulated spectrum to the all-zero spectrum, generating the manipulated spectrum with the settings of the original spectrum.

In gamma spectrometry, it is possible that emissions are not measured at the exactly correct position along the energy axis in the spectrum. Small deviations were corrected before this procedure making use of the positions of the peaks at 511 keV and 1461 keV, which were present in every whole-body spectrum. The offset and slope of the linear energy calibration were calculated from the centroid channels of these 2 peaks, which were read as follows:

- analyze *filename* /seq=*analysis_sequence*.
- where *analysis_sequence* is the filename of an analysis sequence file that contains a peak-search analysis.
- The offset and the slope were transferred to the spectrum as follows:
- pars *filename_original* /ecoffset=*offset* /ecslope=*slope* /ecquad=0.
- The single abundances were automatically read from the nuclide library file using a Genie 2000 report, which was text-processed in the Python script:
- report *filename* /template=c:\genie2k\ctlfiles\liblis.tpl /section=librarylist /screen.
- where *filename* is the filename of the spectrum. This report contains abundances at every emission from the nuclide library, no matter if a peak was identified at the respective energy.
- The single efficiencies were calculated from the efficiency curve depending on the type of curve applied during the calibration (linear, empirical or dual). This allowed a calculation of the efficiency at any energy, no matter if a peak was detected at that energy. The type of efficiency curve was read using the following command:
- getpars *filename* /efftype.
- and its parameters were read depending on the efficiency curve type such as:
- getpars *filename* /dcalfac1,
- where dcalfac1 is one of several parameters defining an empirical calibration curve. For other types of efficiency curves (linear, dual), other parameters exist.
- The combined efficiency was inputted into Genie 2000 by setting the type of the calibration curve to dual (because of the simpleness of its calculation), the crossover point, where the high-energy part of the dual calibration curve starts, to an energy of 0 keV, the parameter dhcalfac1 to the natural logarithm ln_eff of the calculated efficiency *η*_combined_ and all other parameters dhcalfac*i* (*i* = 2…10) to zero:
- pars *filename* /efftype=dual.
- pars *filename* /crossover=0.
- pars *filename* /dhcalfac1=*ln_eff*.
- pars *filename* /dhcalfac*i*=0.

This sets the efficiency over the entire energy range uniformly to *η*_combined_. It must be noted that this restricts the evaluation of the spectrum to the specific nuclide of interest.

The uncertainty $u\!\left({\eta }_{\text{combined}}\right)$ was entered into the evaluation applying the parameters herrmat*i* by

- pars *filename* /herrmat1=*rel_uncertainty_squared*.
- pars *filename* /herrmat*i*=0 (for *i* from 2 to 19).
- where *rel_uncertainy_squared*$$={u}_{\text{rel}}^{2}\!\left({\eta }_{\text{combined}}\right)$$.

## Appendix B: Combination of spectra in the Genie 2000 software

The scaling of the spectra according to Eq. 10 was conducted as follows:

- strip *filename_spectrum1 filename_copy* /factor=1.0.
- strip *filename_spectrum1 filename_copy* /factor=–*scaling*.

where *scaling* is the scaling factor for the higher-efficiency measurement, *filename_spectrum1* is the filename of the spectrum that shall be scaled and *filename_copy* is the filename of a copy of this spectrum. It must be noted that with small channel contents this can lead to an underestimation of counts when non-integer channel contents are rounded down. This can be amended by evaluating the channel contents after the scaling and comparing it with those before the scaling with a subsequent manipulation of single channels as shown in Appendix A (spectrum conversion from the Toolkit format), by which the missing counts are added to these channels.

This spectrum is added to the other spectrum as follows:

- strip *filename_spectrum1 filename_spectrum2* /factor=–1.0.
- The measurement time of the combined spectrum is set with this command:
- pars *filename_spectrum1* /elive=*measurement_time*.

## Appendix C: Calculation of the combined uncertainty of completely correlated quantities

The general equation for the calculation of the combined uncertainty of a quantity *z* that is calculated from 2 possibly correlated quantities *x* and *y* is:

C.1 $$\eqalign{ {u}^{2}\!\left(z\right)&={\left(\frac{\partial z}{\partial x}\right)}^{2}{u}^{2}\!\left(x\right) \cr &\quad+{\left(\frac{\partial z}{\partial y}\right)}^{2}{u}^{2}\!\left(y\right) \cr &\quad+2r\!\left(x,y\right)\frac{\partial z}{\partial x}\frac{\partial z}{\partial y}u\!\left(x\right)u\!\left(y\right)}$$

where *r*(*x*,*y*) is the correlation coefficient between *x* and *y* (Joint Committee for Guides in Metrology 2008; Section 5.2.2, reduced to 2 input quantities).

Farrance and Frenkel (2021) provide a short overview over the calculation in the case where *z* = *x* + *y*. However, in many cases the model of evaluation is *z* = *x*/y such as in the calculation of *η*_*i*, rel_ = *η*_*i*_/*η*_1_ for Eq. 8. In the following, the calculation of the combined uncertainty for such a model of evaluation is explicated:

C.2 $$\frac{\partial z}{\partial x}=1/y$$

C.3 $$\frac{\partial z}{\partial y}=-x/{y}^{2}$$

C.4 $$\begin{aligned}{u}^{2}\!\left(z\right)&= {\left(1/y\right)}^{2}{u}^{2}\!\left(x\right)\\&\quad+{\left(-x/{y}^{2}\right)}^{2}{u}^{2}\!\left(y\right)\\&\quad+2r\!\left(x,y\right)\left(1/y\right)\left(-x/{y}^{2}\right)u\!\left(x\right)u\!\left(y\right)\\&={\left(x/y\right)}^{2}{u}^{2}\!\left(x\right)/{x}^{2}\\&\quad+{\left(x/y\right)}^{2}{u}^{2}\!\left(y\right)\!/{y}^{2}\\&\quad-2r\!\left(x,y\right){\left(x/y\right)}^{2}u\!\left(x\right)\!/x\,u\!\left(y\right)\!/y\\&={z}^{2}\left({u}_{\text{rel}}^{2}\!\left(x\right)+{u}_{\text{rel}}^{2}\!\left(y\right)-2r\!\left(x,y\right){u}_{\text{rel}}\!\left(x\right){u}_{\text{rel}}\!\left(y\right)\right)\end{aligned}$$

In the case of ${\eta }_{i\text{, rel}}$, the 2 efficiencies *η*_*i*_ and *η*_1_ are typically gained from the same efficiency calibration. The uncertainty of an efficiency in alpha and gamma spectrometry is typically governed by the certified relative uncertainty of the activity of the calibration standard, leading not only to a complete correlation of *η*_*i*_ and *η*_1_ but also to an equality of their relative uncertainties also if their values are different. The first point results in a reduction of Eq. C.4 to:

C.5 $$\begin{aligned}{u}^{2}\!\left(z\right) & ={z}^{2}\left({u}_{\text{rel}}^{2}\!\left(x\right)+{u}_{\text{rel}}^{2}\!\left(y\right)-2{u}_{\text{rel}}\!\left(x\right){u}_{\text{rel}}\!\left(y\right)\right)\\ & ={z}^{2}{\left({u}_{\text{rel}}\!\left(x\right)-{u}_{\text{rel}}\!\left(y\right)\right)}^{2}\end{aligned}$$

This is identical to Eq. 15 from Farrance and Frenkel (2021) after a replacement of the absolute uncertainties there by relative uncertainties. The second point results in the uncertainty of *z* or ${\eta }_{i\text{, rel}}$ approaching zero as closer the relative uncertainties of the different efficiencies are to equality.
